# Political Intrusions into the International Health Regulations Treaty and Its Impact on Management of Rapidly Emerging Zoonotic Pandemics: What History Tells Us

**DOI:** 10.1017/S1049023X20000515

**Published:** 2020-04-13

**Authors:** Frederick M. Burkle

**Affiliations:** 1. Harvard Humanitarian Initiative, Harvard University & T.H. Chan School of Public Health, Cambridge, Massachusetts USA; 2. Woodrow Wilson International Center for Scholars, Washington, DC USA; 3. Institute of Medicine, National Academy of Sciences, Washington, DC USA; 4. Prehospital and Disaster Medicine, WADEM, Madison, Wisconsin USA

**Keywords:** coronavirus, global public health, globalization, pandemics, Sir William Osler, World Health Organization

## Abstract

For a large number of health care providers world-wide, the coronavirus disease 2019 (COVID-19) pandemic is their first experience in population-based care. In past decades, lower population densities, infectious disease outbreaks, epidemics, and pandemics were rare and driven almost exclusively by natural disasters, predatory animals, and war. In the early 1900s, Sir William Osler first advanced the knowledge of zoonotic diseases that are spread from reservoir animals to human animals. Once rare, they now make up 71% or more of new diseases. Globally, zoonotic spread occurs for many reasons. Because the human population has grown in numbers and density, the spread of these diseases accelerated though rapid unsustainable urbanization, biodiversity loss, and climate change. Furthermore, they are exacerbated by an increasing number of vulnerable populations suffering from chronic deficiencies in food, water, and energy. The World Health Organization (WHO) and its International Health Regulation (IHR) Treaty, organized to manage population-based diseases such as Influenza, severe acute respiratory syndrome (SARS), H1N1, Middle East respiratory syndrome (MERS), HIV, and Ebola, have failed to meet population-based expectations. In part, this is due to influence from powerful political donors, which has become most evident in the current COVID-19 pandemic. The global community can no longer tolerate an ineffectual and passive international response system, nor tolerate the self-serving political interference that authoritarian regimes and others have exercised over the WHO. In a highly integrated globalized world, both the WHO with its IHR Treaty have the potential to become one of the most effective mechanisms for crisis response and risk reduction world-wide. Practitioners and health decision-makers must break their silence and advocate for a stronger treaty, a return of the WHO’s singular global authority, and support highly coordinated population-based management. As Osler recognized, his concept of “one medicine, one health” defines what global public health is today.

## The International Health Regulations (IHR) Dilemma

The World Health Organization (WHO; Geneva, Switzerland) was established to further international cooperation for improved public health. As the directing and coordinating authority on international health within the United Nations (UN) system, WHO inherited specific tasks relating to epidemic control. Specifically, its main mandates include “directing, leading, and coordinating the health response during infectious disease emergencies, working with countries to increase and sustain access to prevention, treatment, and care, and identifying priorities and setting strategies.”^1^ Specific International Health Regulations (IHR), with timely revisions, were conceived to serve as a first step in providing specific and universal medical knowledge of a unique infectious disease outbreak. The WHO also created distinctive rules, regulations, and organizational constraints, such as travel restrictions, examples being the emergence and pandemic potential of HIV/AIDS and the urgency provoked by severe acute respiratory syndrome (SARS) in 2003.^2^ The IHR must immediately notify the WHO of any outbreaks that constitute a public health emergency of international concern. The IHR is also obligated to immediately alert and marshal resources and coordinate all global response efforts.

Whereas the IHRs provide a vital governing framework to limit the spread of disease, serious deficiencies, omissions, gaps, and political resistance began to occur. Gostin and Katz described wide-spread noncompliance to the IHR detailing multiple needed textual and operational reforms, emphasizing that WHO and the IHR “erred at multiple levels during the Ebola epidemic” and WHO failed “to mobilize adequate fiscal and human resources until the epidemic was spinning out of control.”^3^ In 2015, after the Ebola epidemic, I wrote “the intent of the legally binding Treaty to improve the capacity of all countries to detect, assess, notify, and respond to public health threats has shamefully lapsed,”^4^ and that global health security demanded both a stronger WHO and a stronger IHR treaty.

The WHO, sponsored by the UN, currently has two primary sources of revenue: assessed contributions expected to be paid by member-state governments, income, and population; and voluntary contributions provided by member-states and contributions from private organizations and individuals, the latter of which opens the WHO up to being influenced by the highest bidder.

The WHO must exist solely as a treaty-based organization sanctioned by the UN and all its members, not dependent on outside financial assistance to do its work. Health care experts, as Sir William Osler described, must be in charge of all health decisions, monitoring, response, and operational research. They neither abdicate any responsibilities to individual nation-states nor be beholden to them for support.

The bottom line is that the global community can no longer tolerate an ineffectual and passive international response system, nor tolerate the self-serving political interference that authoritarian regimes, nationalism, and populism demand. This remains a highly integrated globalized world when it comes to public health protections. The current coronavirus disease 2019 (COVID-19) pandemic experience leads to only one solution: the WHO must be restructured from top to bottom to remove individual countries from health and public health assessment, decisions, and management. Without political pressure, WHO and the IHR Treaty have the potential to become the most effective partners in crisis response and risk reduction. Practitioners and health decision-makers world-wide must break their silence and advocate for a stronger Treaty and a return of WHO’s singular authority.

## The Legacy of Sir William Osler

The majority of health care providers world-wide practice one-on-one care with their patients. Population-based care has not been emphasized in their training. A major deficiency in global health and the entire IHR process has been the failure to recognize the importance of zoonotic diseases–those diseases that can be passed from animals to humans. World-wide, zoonotic diseases represent 61% of all diseases and an alarming 71% of new diseases. Second only to war, zoonotic epidemics have killed more humans than any other disease. Today, it is known that climate change, overuse of antibiotics, and more intensified farming are thought to also be increasing the rate of zoonotic diseases globally.^5^

My first experience in zoonotic disease was in 1968 during the Vietnam War. That year, South Vietnam witnessed the largest bubonic plague epidemic of the 20^th^ century. Indeed, that outbreak temporarily paused the war on both sides of the conflict and emptied small villages and larger city streets. It prompted me to educate myself on the massive influence zoonotic diseases have on the environment and public health.

For many of my generation of physicians, Sir William Osler remains a crucial role model, famous for his writings and for taking the teaching of medicine out of the classroom to the bedside. Few know that he taught at both medical and veterinarian colleges, advancing the basic knowledge of veterinary pathology and zoonotic diseases, or those that commonly spread from non-human animals to humans. Osler’s work advanced the understanding of today’s infectious disease outbreaks, epidemics, and pandemics. It was a veterinarian, Calvin Schwabe, schooled under Osler’s teachings and now recognized as the father of veterinary epidemiology, who first coined the term “One Medicine” as the science of health and disease in which “differences between humans and animals are not considered.”^6^ Schwabe pointed out that most infectious diseases of humans have an animal origin that incorporated the “inclusion of environmental health, as opposed to simply medical treatment”^7^ into the crucial management of major infectious diseases such as SARS, H1N1, and today’s COVID-19.

In his 1906 book *Aequanimitus,* Osler emphasized that medicine is the “only world-wide profession following the same methods, actuating the same ambitions, and pursuing the same ends.” He emphasized that this “homogeneity” or “solidarity” which physicians world-wide are witnessing today with the COVID-19 pandemic is a quality “not shared by law” or politics, that “allows physicians to practice the same art amid the same surroundings in every country on earth.”^8^ This unity of effort is not seen in other professions, and is witnessed today with the wide support given by medical colleagues from other countries, all cooperating on essential clinical and public health research.

Sadly, Osler died in 1919 at age the of 70 from Spanish Influenza while teaching at Oxford (England). The Spanish Influenza was also called “swine flu” because it allegedly jumped from live pigs to humans, killing one-quarter of the world’s population.^9^ The capacity for swine flu to survive and to initiate a second pandemic in 2009 was possible because it thrived and spread as a new mix of genes from swine, birds, and human flu viruses. We now live in an age of epidemics and pandemics. Predictably, and in its own time, the swine flu will reemerge once again.

## Origins of Global Public Health

Modern day scholars have taken the “One Medicine” concept and advanced it into the “One Health Initiative,” a movement that seeks to “forge greater collaboration between the health disciplines,” advocating for multidisciplinary efforts to improve global health in general. It became a globally shared concept and world-wide strategy for expanding interdisciplinary collaborations and communications in all aspects of health care for humans, animals, and the environment. The synergism accelerated “biomedical research discoveries, enhancing public health efficacy, expeditiously expanding the scientific knowledge base, and improving medical education and clinical care.”^10^ When properly implemented, the “One Medicine” concept would help protect and save untold millions of lives in present and future generations.^11^

This concept eventually incorporated specific expertise in biohazard events, food and water safety, vector-borne diseases, established and emerging zoonotic diseases, herd health, foreign animal risks, and public health issues such as antimicrobial drug resistance.^12^ This would come to define One Health advocates and practitioners of the future, and today defines what we now refer to as the operational elements of “global public health.”

## Life and Death of Globalization

Beginning in the 1970s, major economic and political changes occurred when economically leading Western countries developed businesses in third world countries, a process referred to as “globalization.” With it came the realization that the economy was a major force behind the setting of public policies, including health policies. Increasingly, the process revealed that the power of governments to shape national policy was, in many cases, being considerably limited and diminished by an increasingly competitive international economy where some countries impacted by globalization either thrived or collapsed.^13^

Global health experts, and those focusing on humanitarian and crisis management, who were excluded from any cross-cultural economic debates, closely watched from afar how and where public health infrastructure and protections in water, shelter, food, and availability of health services would either benefit or suffer from globalization. Too often, local public health and the global health priorities they impacted took a backseat to economic demands resulting in “weakening of life-supporting systems,” specifically “altered composition of the atmosphere, land degradation, depletion of terrestrial aquifers and ocean fisheries, and loss of biodiversity.”^14^ These are elements known today that can lead to acquiring and spreading of epidemic infections such as SARS, H1N1, and Influenza. Health and public health were never at the same globalization negotiating table, but were more often silently relegated to a catchup role that tried to mitigate the impact on health caused by increasing globalization. With increasing globalization and speed of transportation, infections rapidly began migrating across borders. Yet WHO, now equipped with improved telecommunications, developed an increased capacity to readily detect emerging epidemics, a major improvement never before available with previous epidemics and pandemics.

An encouraging aspect of globalization was the increasing number of the millennial generation who studied abroad and worked on various humanitarian missions. As a result, they began seeing themselves less as nationalists and more as global citizens.^15,16^ However, with the recent rise and dominance of authoritarian regimes and populism, globalization has essentially noticeably faded and is rapidly becoming a non-entity. Both the word “globalization” and its concept have disappeared under a coordinated false narrative campaign promoted by autocrats and rising nationalist state movements.^17,18^

Ghitia contends that “modern-day would-be dictators don’t overthrow another government. What they do is take over the system of government.” She emphasized that their methods are more gradual, “manipulating the democratic norms, wearing them down to a thin shell that contains only the wrecked remains of democracy.”^19^ By the time most people realize what happened, it is too late to push back. I talked to an investor once active in the globalization movement, asking what was going to happen with the large number of desperately needed public health infrastructure projects. His response was, “only if they can show us a profit.”

## Increasing Threats that Enable Pandemic Spread

In past decades, human population densities were much too low for viral illnesses to widely occur and outbreaks were, more often than not, driven almost exclusively by natural disasters, predatory animals, and prolonged wars. Globally, zoonotic spread occurred simply because the human population has grown in numbers and become more dense. The spread was enhanced and accelerated by rapid unsustainable urbanization, biodiversity loss, climate change, and its extremes. This has resulted in producing further viral engagement with an increasing number of a new vulnerable populations suffering from chronic deficiencies in food, water, and energy.

The current SARS COVID-19 transmission that flourished in wet market animals, whether it be a bat or civet, spread easily to the human-animal, a perfect host. The chaos created by the rapid spread of COVID-19 has created an unprecedented opportunity for state-sponsored disinformation. Probably the most infamous infectious disease disinformation incident was the KGB’s “Operation Infektion” in the 1980s, which blamed the United States for the creation and spread of HIV. Although the Union of Soviet Socialist Republics (USSR) conceded in 1992 that the KGB had instigated and perpetuated the myth, considerable damage was done, most importantly global distrust of the “official narrative” which fed into claims that HIV does not cause AIDS and distrust that the anti-retroviral used to treat HIV was useless, resulting in more than 330,000 preventable deaths.^20^ What continues today is a Russian autocratic regime that still places greater emphasis on false epidemic narratives than solving its own fast-growing global rates of HIV/AIDS and tuberculosis (TB).

Russia and China are exploiting both real-life mistakes and weaknesses in the information space to control and modify the narrative with impacts on geopolitics and national security. Spreading conspiracy theories from China, Russia, and the United States is rampant, all systems designed to deflect responsibility for their bureaucratic failures. China is now seeking to blame the United States for COVID-19 claiming, “further evidence that the virus originated in the US” and was planted in China by the US Army. Russia is sowing divisions between and within Western countries to undermine public confidence in government competence and integrity.^21^

The WHO, despite having in-hand evidence to the contrary, failed to properly contain the COVID-19 pandemic. China’s gross denial and failure to investigate and alert other nations is inexcusable. Moreover, its malignant behavior toward clinicians and researchers who warned the government of the outbreak, when the virus was first known as far back as October of 2019, is equally inexcusable. Yet the February 16-24, 2020 “Report of the WHO-China Joint Mission on Coronavirus Disease 2019” singularly praised China’s response as the best source of medical technology to deal with the pandemic. China then declared that their singular success in controlling the pandemic should qualify them to take over the WHO.^22^

While the European Union and the United States struggle to control the COVID-19 pandemic, WHO fully supports China’s “One Belt, One Road” initiative across Africa to improve the economy of the continent. However, the lessons from globalization prove that economic prosperity alone cannot be achieved when huge knowledge and capacity gaps exist in health systems, especially public health and health information systems. There is a need for public health initiatives aimed at strengthening the health systems beyond sovereign borders to influence global geo-economics.^23^ Whereas WHO has fully supported this initiative with claims that China is investing in “people’s health outside its border,” the deplorable cover-up, response, and management of COVID-19 for many months before it was known to the world questions whether China is up to the public health challenges it claims in Africa, or fully understands the vital connections economic development has with public health. China claims that ruling Africa’s economy is a necessary prelude to the “next phase of globalization.”^24^

Ever since WHO first announced the presence of clusters of unknown pneumonia on December 31, 2019, an alarming concern has surfaced that WHO has become beholden to influential countries for funding support, giving wealthy UN members, especially China, support and influence both before and during the coronavirus outbreak. For example, WHO’s position regarding China has renewed a longstanding debate about whether WHO, founded 72 years ago, is sufficiently independent to allow it to fulfill its purpose.^25^ Critics raised questions concerning WHO’s response over how “China’s sway over the WHO is its success in blocking Taiwan’s access to the body, a position that could have very real consequences for the Taiwanese people if the virus takes hold there.” Others cite that WHO “downplayed the harsh control of medical whistleblowers,” and the critical delay in revealing COVID’s presence, and further argue that WHO is “overly bureaucratic, bizarrely structured, too dependent on a handful of major donors, and often hamstrung by political concerns.”^26^

With the COVID-19 crisis, “the state of politics and geopolitics has exacerbated, not stabilized, the crisis.”^27^ This applies to many countries, especially China, the United States, Japan, Cambodia, Iran, and South Korea. Authors cite former WHO consultant Charles Clift who observed, as have many former insiders, that WHO “is too politicized, too bureaucratic, too dominated by medical staff seeking medical solutions to what are often social and economic problems, and too timid in approaching controversial issues, too overstretched, and too slow to adapt to change.” He added that WHO, being “both a technical agency and a policy-making body, that excessive intrusion of political considerations in its technical work can damage its authority and credibility as a standard-bearer for health.”^25,28^

United States’ President Trump has not done better in what must be a coordinated world response. His idea of “America first and national populism” is against everything that we believe in global health.^29^ In January 2019, China made available the “genome” of this mysterious new virus in hopes of producing the first diagnostic test for the disease, but the United States declined to use the WHO test even temporarily as a bridge until the US Centers for Disease Control and Prevention (CDC; Atlanta, Georgia USA) could produce its test. This action remains a perplexing question and the key to the Trump administration’s failure to provide enough tests to identify the coronavirus infection, needlessly slowing the critical domestic testing process and surveillance.^30^ Additionally, President Trump’s reliance on the validity of his “hunches,” claimed WHO’s mortality rate was “false,” irresponsibly valuing his “best guesses over scientific analysis.” This has led to a “false sense of security that endangers public health.”^31^

Both China and the United States have public health infrastructure and service deficiencies that have gone unattended for decades. Public health infrastructure in the United States makes up only three percent of health care spending focused on prevention and public health, while 75% of health care costs are related to preventable conditions.^32^ China chronically suffers low public health standards in toilets, restaurants, hospitals, and meat markets; and the United States has 50 states and 55 very different health department ratings.^33^ As an example, during the COVID-19 pandemic, Mississippi, which rates last in public health infrastructure, has created confusion with many of its mayors claiming the need for curfews and closing of businesses, only to be over-ridden by the State’s governor.^34^

## The Only Solution

The WHO must exist solely as a treaty-based organization sanctioned by the UN and all its members. It cannot be dependent on outside financial assistance to do its work. The unique characteristics of propagating zoonotic diseases must be better known by both the medical profession and governmental decision makers. Health care experts, as Osler described, must be in charge of all health decisions, monitoring, response, and operational research. They cannot abdicate any responsibilities to individual nation-states nor be beholden to them or well-financed donors for support.

Current disaster taxonomy describes diversity, distinguishing characteristics, and common relations in disaster event classifications. The impact of compromised public health infrastructure and systems on health consequences defines and greatly influences how disasters are observed, planned for, and managed, especially those that are geographically wide-spread, population-dense, and prolonged.^35^ The One Health concept helps to set the path forward for a solution based on local grassroots coordination, and a bottom-up capability driven by medical, veterinary, and public health practitioners. This must include rapid, networked information sharing and the use of multiple expert disciplines to mitigate an outbreak.

Lastly, public health and public health infrastructure and systems in developing countries must be seen as strategic and security issues that deserve international public health resource monitoring. This must cover the entire disaster cycle from prevention, preparedness, response, recovery, and rehabilitation.^36^ All six WHO Regional Offices must have similar multidisciplinary professional assets in support of zoonotic sciences. As Osler might declare today, “There is so much more we need to know!”
